# *“They gave us the right to choose.”* A qualitative study of preferences for differentiated service delivery location among recipients of antiretroviral therapy at Lighthouse Trust in Lilongwe Malawi

**DOI:** 10.1371/journal.pone.0296531

**Published:** 2025-02-06

**Authors:** Lisa Orii, Kate S. Wilson, Jacqueline Huwa, Christine Kiruthu-Kamamia, Odala Sande, Agness Thawani, Astrid Berner-Rodoreda, Evelyn Viola, Hannock Tweya, Petros Tembo, Wapu Masambuka, Richard Anderson, Caryl Feldacker

**Affiliations:** 1 Paul G. Allen School of Computer Science & Engineering, University of Washington, Seattle, Washington, United States of America; 2 Department of Global Health, University of Washington, Seattle, Washington, United States of America; 3 Lighthouse Trust, Lilongwe, Malawi; 4 Faculty of Medicine, Heidelberg Institute of Global Health, Heidelberg University, Heidelberg, Germany; 5 International Training and Education Center for Health (I-TECH), University of Washington, Seattle, Washington, United States of America; Vellore Institute of Technology, INDIA

## Abstract

Some differentiated service delivery (DSD) models for antiretroviral therapy (ART) allow stable recipients of care (RoC) to receive multi-month ART drug refills and complete rapid reviews in community sites. As DSD options expand across sub-Saharan Africa, RoC’s preferences and perspectives on community-based DSD versus clinic-based care models warrants attention. Lighthouse Trust (LT) implements DSD services for the Ministry of Health in Lilongwe, Malawi, including a community-based ART service delivery model that complements it traditional, clinic-based care. In this qualitative study, we explore reasons why RoC enrolled in LT clinics and eligible for DSD chose clinic-based ART services or a Nurse-led Community-based ART Program (NCAP) that reaches clients in established community peer support groups. We conducted eight focus group discussions (FGDs) among LT RoC: four FGDs among NCAP groups and four clinic-based FGDs (2 per setting) to explore opinions, preferences, and perceptions about ART service delivery. FGDs were recorded in Chichewa, translated and transcribed into English for thematic analysis. Findings were discussed with LT and NCAP teams to ensure results resonated with their personal experiences. Sixty-three participants took part in FGDs. Many findings were similar across care model. Across both NCAP and clinic FGDs, RoC were pleased with the care quality and appreciated the convenience of integrating their appointment visits at their chosen care model into their daily lives. Across FGDs, RoC also appreciated the quality of care, the respectful provider-to-patient interactions, and the attention to privacy at community and clinic sites. RoC in both clinic and NCAP care models expressed satisfaction with their chosen care model and preferred that choice over alternative options and locations, some noting their willingness to travel far to access LT’s high quality of clinic-based care. Privacy protection was an important consideration for choosing care models. At LT clinics, RoC highlighted the importance of physical separation between LT’s HIV-specific service site and other care services. In NCAP, RoC expressed that their choice of care model was reinforced by the sense of mutual support they received through NCAP peer support. These findings suggest the importance of offering personal choice to RoC on care model and selection of DSD options to support their ongoing engagement in care.

## Introduction

To reach the global target of eliminating new infections by 2030 [1], health systems in sub-Saharan Africa (SSA) must adapt existing resources to increasing numbers of recipients of care (RoC) on lifelong HIV care and treatment. Malawi has 950,000 adults aged 15 and over living with HIV (7.1% prevalence), of whom 92% receive antiretroviral therapy (ART) [1,2]. Since 2017, many countries have adopted differentiated service delivery (DSD) models, where RoC with suppressed viral load and adherence to antiretroviral therapy (ART) adherence can access alternative care models. DSD models are usually community-based and are provider or RoC-led, often with peer supporters from that community to promote ART adherence and offer social support [3]. DSD models are designed to be more client-centered and to reduce the burden on both clinic staff and healthcare systems in comparison to standard clinical care models. However, there is limited evidence on comparative feasibility, quality, effectiveness, or cost between different models or settings [3,4]. One randomized trial in Zimbabwe found that retention and viral suppression outcomes were similar between RoC receiving 3-month refills from community sites compared to those receiving 3-month refills at clinics, suggesting that community-based ART models are as effective as clinic based extended refills [5]. Recent qualitative studies explored RoC and provider experiences in HIV care using DSD models. RoC in community-based models reported that fewer clinic visits reduce their clinic wait times and transportation costs [6–8], increase peer support [9,10] and improve treatment adherence [11]. By contrast, RoC receiving DSD models at facilities in Zimbabwe reported preferring clinic-based care to community-based refill options because the clinics were near work [6] or they feared stigma and discrimination through inadvertent disclosure of their HIV status if they collected ART at a community venue [6,7,9,12,13]. Healthcare providers also reported that RoC’s concerns around confidentiality and unwanted disclosure of HIV status posed barriers to using clinic-based DSD models [6]. It appears that RoC may have different reasons for using care models offered at a clinic compared to community-based DSD models. Perspectives from RoC in routine HIV care settings with access to both clinic and community-based DSD models, are lacking.

Given national efforts to expand DSD options in SSA to improve RoC-centered care using clinic and community models [14], RoC preferences, opinions, and perspectives on care models warrant further attention. To address this gap, we conducted a qualitative study among RoC to explore opinions, preferences, and perceptions about using a clinic-based model and a community-based DSD model at Lighthouse Trust (LT) in Lilongwe, Malawi. LT is a large, public ART provider with over 38,000 RoC in two urban clinics and 120 community-based care sites in Lilongwe. This study may inform service delivery considerations in Malawi and other low-resource ART programs in the region.

## Methods

### Setting

Lighthouse Trust (LT) supports the ART program of the Malawi Ministry of Health (MoH) and provides integrated HIV prevention, treatment, care, and support services in five high-volume Center of Excellence clinics across the country. LT is widely recognized for its innovations and efforts to improve the quality of care over two decades of HIV-related service delivery [15–22]. In urban Lilongwe, LT runs two, large flagship clinics with 25,148 RoC at Martin Preuss Center (MPC) at Bwaila Hospital and 12,853 RoC at the Lighthouse Clinic (LH) at Kamuzu Central Hospital. At these two flagship clinics, viral load suppression at 12 months on ART is higher than 90% according to routine monitoring and evaluation data. LT also supports care at nine peri-urban satellite sites in Lilongwe district. LT implements the same training, policy, and practices at all sites, including use of the Malawi MoH’s real-time, point-of-care electronic medical record system (EMRS). LT implements several care models. Clinic-based models include expanded early and late service hours, adolescent-friendly programs, integrated family planning services, and non-communicable disease care. LT’s community-based DSD model is the Nurse-led Community-based ART Program (NCAP). In NCAP, community-based nurses provide ART services to eligible RoC referred from all LT clinical settings in Lilongwe. RoC from LT clinics may be referred to NCAP if they meet the following criteria: 1) at least 18 years of age; 2) clinically stable; 3) on ART for at least 6 months; and 4) member of a peer support group. All NCAP clients previously received services at LT clinics. NCAP operates in the community during routinely scheduled peer support meetings held in churches, community centers, schools, or other gathering places according to the peer group preference. RoC meet with a community nurse who visits that location with an established peer support group every 3–6 months. In 2023, over 5,195 RoC in 120 peer groups in Lilongwe were enrolled in NCAP and reported viral load suppression among NCAP RoC on treatment was ~ 95%. NCAP receives high levels of client satisfaction and has healthcare worker support for expansion [7].

### Study design and population

As part of a larger qualitative study on data privacy and security in both community and clinic settings [23], we conducted eight focus group discussions (FGDs) with RoC enrolled in clinic-based care models and the community-based NCAP model from September 7, 2022 to October 14, 2022. FDGs were not conducted in the satellite sites. The FGD facilitator was a trained Malawian LT qualitative researcher who speaks both Chichewa (the most common language in Malawi) and English. We used purposeful sampling at two flagship clinics and four NCAP care locations to enroll 63 RoC in 8 FGDs of 6–10 people each—four FGDs with RoC who received clinic-based care (2 at LH and 2 at MPC) and four FGDs with RoC who received care through NCAP. We determined that eight FGDs was sufficient to reach theoretical saturation of key themes [24]. LT staff providing care for both models screened RoC for study eligibility: 1) age 18 and above; 2) at least a primary education; 3) written voluntary consent to FGD participation; and 4) permission to audio record the discussion. FGDs were conducted at the location of participants’ routine care model, either the clinic or NCAP site.

### Data collection

Four FGDs took place at two flagship clinics and four NCAP sites. All FGD participants signed confidentiality agreements to not share information discussed outside the group and gave permission to have sessions audio recorded. Each FGD included seven to nine participants. FGDs were conducted using a semi-structured discussion guide. The guide explored RoC’s opinions, preferences, and perceptions about using clinic-based and community-based care models. In this study, we focus on reasons for accessing ART services at their chosen care model, including HIV-related stigma, and consideration for alternative care locations. A Malawian qualitative researcher facilitated all FGDs in Chichewa. FGDs were audio recorded and lasted about an hour. After each FGD, the facilitator translated and transcribed the FGD into English and completed a debrief report summarizing key points, participants’ openness, and group dynamics. The research team reviewed and discussed all debrief reports.

### Analysis

FGD transcripts were analyzed using thematic analysis, combining deductive and inductive steps [25]. Two team members (located in the United States and who did not conduct the FGDs) conducted a detailed reading of the full-length transcripts and developed an initial codebook restricted to the questions about care model preferences, including concepts related to stigma, in ATLAS.ti v.8. A second team member reviewed the code application and resolved disagreements through discussion with the main analyst. The analyst team shared initial results and coding with the LT study team members for their inputs before finalizing the code list.

### Ethical considerations

This study was approved by the University of Washington Institutional Review Board (STUDY00013936) and the National Health Sciences Research Committee of Malawi (# 2968). All participants provided written informed consent. All participants were compensated with K10310.00 (approximately 10 USD) for their time. This study was conducted in accordance with the LT team and researchers whose primary interest is to improve the quality of care of RoC.

### Researcher positionality

The author group consists of international researchers from a US academic institution and local researchers embedded in the HIV program setting at LT. The group benefited from in-depth local knowledge and experience of HIV studies across various geographic settings. All authors used an iterative process of data review and reflection [26], including group discussions of preliminary results and discussion on how individual perspectives may influence data interpretation. This process is designed to strengthen the study’s methodological rigor, transparency, and inclusion throughout the research process, especially during data collection, analysis, and results reporting.

## Results

Overall, 63 FGD participants took part in this study. The median age was 47 (IQR 41–56), 81% were female, 51% received care from NCAP and 49% from clinics (Table 1). Overall, participants from both NCAP and the clinics reported positive experiences with their chosen care model. We identified four themes about participants’ reasons for choosing their care model: (1) convenience including integration into daily activities and efficiency; (2) high quality of care; (3) assurance of privacy; and (4) sense of mutual support. We compare findings from clinic and NCAP discussions.

**Table 1:** Gender and Age Distribution of FGD Participants.

Site of Care	Participants per Group (n); n% Female	Mean Age
*Clinic FGD*		
1	8; 6 females (75%)	40
2	7; 5 females (71%)	41
3	8; 6 females (75%)	34
4	8; 5 females (63%)	56
*NCAP FGD*		
1	8; 6 females (75%)	54.5
2	7; 7 females (100%)	48
3	8; 7 females (88%)	50
4	9; 9 females (100%)	45
Total	63 (81% female)	47

### Recipients of care want convenience

The convenience of integrating appointment visits into daily routines influenced participants’ choice of care model. This sentiment was expressed more strongly among participants from NCAP than the clinics. Short travel distance was a major factor that influenced participants’ choices.

*“I am thankful for this arrangement where we must be getting medication in the community. Sometimes we leave some activities in the community, we come here and get medication and attend to activities like funerals within the community. In addition, when we are sick, we can get medication here when the providers visit the community...Bringing the community ART program has helped us a lot. We can leave beans boiling on fire, come here and get back home, and still find the beans boiling. We are very grateful.”* – P5-NCAP2

For some clinic-based participants, short distance to the urban clinic and extended hours of operation enabled them to integrate clinic visits into their work schedule.

*“I like this clinic because it is near to my home, as such I do not struggle with transport. In addition to that, they open the clinic early in the morning. I can go back to work once I collect my medication.”* – P1-Clinic2

However, not all clinic-based RoC can visit the clinics so easily. Many NCAP participants said that the distance, travel time, wait time, and costs associated with accessing clinic-based services was a factor in opting for NCAP, noting a reduction in their travel time as a result.

*“We have different ways of getting transport money, some of us struggle to find money. By bringing the medication right in the community, they reduced the distance. We consider it to be a great thing because it helps to reduce the distance.” –* P3-NCAP2

In addition, participants reported that they no longer needed transport, and therefore transport fees, by choosing the NCAP model. The NCAP location made it easier to attend support group meetings and receive consistent ART.

*“We like getting our medication at this place because we save transport money. In the past, one would fail to report to the facility because they do not have money for transport, and that made them not to be adherent to medication. Bringing the community program made us not to miss appointment dates and to be taking medication properly.”*– P8-NCAP3

### Recipients of care value high quality of care

In FGDs from both clinics and NCAP, RoC expressed that providers respected and cared for them kindly, which affirmed their choice of care model. At the clinics, participants noted that nurses offered adherence counseling and emotional support to encourage them to live healthy lives and accept their serostatus.

*“Since I started taking medication. I am encouraged especially because of the counseling that is provided at Lighthouse so that one accepts their status. They provide counseling on being adherent and other issues that make one encouraged and be healthy [as it was before].”* – P1-Clinic3

Clinic-based participants also compared the quality of care at LT clinics to that of other hospitals or clinics, making informed choices on which clinic to attend: *“There are some sites where one would be disappointed because they have been shouted at. Even though I live far from here, I make sure that I report to the [Lighthouse] clinic”* (P3-Clinic2). Distance and cost of care was therefore not a major consideration for some RoC when choosing care model.

*“Even though I live far from here, I heard that they manage the disease very well at this clinic. When I was referred here, I noted that there is expertise at this clinic. As I continued collecting medication here, I noticed that they have good reception. During my first visit, I noted that there is respect and love for the client, this is different from what I experienced in other sites. It made me happy, and I decided that even though I live far from the clinic I will be reporting to this clinic.”* – P6-Clinic2

NCAP participants reported that community nurses were more respectful and friendly as compared to clinic providers. This observation reflected a common perception that NCAP providers were interested in the lives of RoC, which encouraged RoC to openly share health issues with nurses. One participant characterized NCAP providers by noting that, *“they warmly welcome us; they are happy and interested in us. In addition to that they are attentive when we are sharing our issues with them, they provide the right kind of support”* (P6-NCAP4).

Providers offering responsive and appropriate care that was easy to understand was seen as another incentive for staying in NCAP. Specifically, participants appreciated how NCAP providers offered referrals and reminders for other preventative healthcare.

*“Our providers provide services appropriately, whenever we have issues in addition to the medication that we get, they act so fast to refer one to the clinic where they can get assistance. Whenever they have something to share with us, they make sure that they deliver the information in a proper manner so that we can understand. We are so happy with the doctors who come here.”* – P4-NCAP2

### Recipients of care want their privacy protected

Privacy was an important consideration for all participants in choosing their care model. Clinic-based participants chose longer travel times and distance to receive care at LT to avoid encountering people from their communities. The primary reason was fear of unwanted disclosure of HIV status and discrimination.

*“I also come from far, but I still come here because I believe my privacy can be maintained because it is far from my home. I did not meet people from my community when I came here. There are others who discriminate against others once they learn their HIV status. When I came here my confidentiality is maintained.”* – P4-Clinic2

Many participants reported that they felt reassured that they would not be seen by people they knew because both LT ART flagship clinics are in a separate section of the hospital.

*“I am happy, nowadays. Those of us who are positive and on treatment are stigmatized, we are seen differently. I am happy because they separated the clinic, it isolates us from them, and they do not know where we go and what we do there. It makes me happy to come to this clinic.”* – P5-Clinic1

Within the LT clinic, participants felt that privacy and confidentiality were well-maintained, noting that providers ensured that conversations were held in a private room.

*“When we get into the nurse’s room, they tell us to close the door. It is only the client and the nurse in the room. So, whatever we discuss is confidential, it remains between the nurse and the client. They maintain our confidentiality.”* – P2-Clinic3

Several NCAP participants also reported they had more privacy through service delivery at NCAP model sites. The LT clinics are located near general health services on the main hospital campus, where there was greater potential to encounter someone from the community they knew, including guardians of other RoC, who might disclose their status to others.

*“Sometimes it happens that one has sent a guardian, and you meet them [referring to the guardian]. It then becomes embarrassing when you meet at home. If the person that you met is not able to keep it to themselves then they will tell other people that you met at the clinic and that you are taking medication. That made others not to report to the clinic with fear that they will meet other people who know them.”* – P8-NCAP3

RoC who chose NCAP also explained that physical separation of spaces at NCAP sites enhanced the sense of privacy.

*“When the nurses get here, there is a distance between them and the recipients of care, even if one looks at them there is nothing that they can hear. There was another place where we had a side room, where one would not hear what is being discussed no matter how hard they try to pay attention. There is a distance between other recipients of care and the nurse.”* – P3-NCAP4

However, some RoC chose to remain in clinic-based care because of the risk of unwanted disclosure through NCAP participation.

*“In the past there were groups and once you join you were connected to a smaller group where you were visited. But there were the same people [from the group] who were disclosing other people’s status, yet this is confidential. A lot of people thought that those groups were not good, and a lot of people decided to drop out.”* – P7-Clinic4

### Choice of care model is reinforced by sense of mutual support

NCAP participants appreciated the peer support they gain through their care model - a sentiment that was expressed exclusively by NCAP participants. Most NCAP participants reported that the community group model enabled them and their peers to stay better engaged in care compared to at the clinics. Participants shared how they supported each other to attend community visits, including volunteering to call or visit other RoC to remind them to meet.

*“We are very comfortable. We also encourage each other as clients who report to the community to get medication. We encourage each other that if we have noticed one of our friends is sick and is not aware of the main issue, we visit them. Though we are not volunteers, we tell them the facility where they can get help. It is good to be open.”* – P4-NCAP2

The mutual support between NCAP RoC also enabled them to encourage one another to seek care for other health issues.

*“We are happy to be getting medication in the community. It is a good thing because we encourage each other about health and some of the issues that we face. Unlike at the clinic like [X] or [Y] where there are different people, and it is hard to be free to interact. Here [in the community] we meet too often, and it makes it easy to encourage each other.”* – P6-NCAP2

NCAP participants also felt that they supported the nurses’ role in care delivery. NCAP RoC and group leaders worked closely with community nurses to track other RoC who missed their scheduled appointments, which likely helps to reduce the number of defaulters.

*“We work with the providers, and this has reduced the number of defaulters…We are able to know the appointment dates of the clients and we are able to remind them, when they do not show up to the community distribution point. We follow them up and bring them here. It is unlikely to have defaulters and we have reduced the workload for the providers.”* – P7-NCAP2

The collaboration between the group leader and the nurses also allowed volunteers to deliver medication to RoC who, for various reasons could not collect them from the nurse, thus encouraging continuation of care: *“I am thankful because when we forget to get the medication, they visit us at home and give us medication”* P4-NCAP3.

## Discussion

In this qualitative study to explore RoC preferences for community- or clinic-based ART care models, we found similar reasons for why both groups chose their model, reinforcing the value of continuing to offer multiple care models. Convenience, privacy, and quality of care were perceived as facilitators for both groups in selecting their preferred care model. Those in NCAP also commented on the role of peer support in improving their care experience. ART service choice offered RoC with the flexibility to choose the care model that best met their needs, likely boosting satisfaction that could improve motivation to remain in care. Comparing perspectives of RoC on ART services in two care models across different locations run by the same clinical team enhances the strength of the results. These findings suggest several areas of consideration.

RoC in both models were concerned with privacy and the potential for stigma, giving them a choice to remain in their care setting to avoid HIV-related stigma. While DSD is scaling up, frequently to community settings, previous studies suggest fear of unintended disclosure as a barrier to uptake of community-based DSD models [6,7,9,12,13,27]. In our study, some clinic-based RoC compromised travel and time concerns to receive care at LT, rather than in the community, to avoid being seen receiving HIV-related services. The separation of LT ART clinics from other sections of the hospital enhanced clinic-based RoC’s sense of privacy. While this may be unique to the operation of LT, physical separation of ART clinics from the main facility likely contributed to the sense of privacy and comfort. Interestingly, participants did not express disclosure concerns with peer supporters (who are community members) in the NCAP model, although this topic merits further exploration in community-based DSD models. These results again reinforce the need for diverse care setting options.

RoC appreciated care models that accommodated their daily routines, underpinning the importance of designing care models that address the needs and preferences of RoC [13,28,29]. RoC from both clinics and NCAP recognized the importance of integrating appointment visits into their daily routines. Clinic-based participants appreciated that they could easily fit their visits into their work schedules. For NCAP RoC, the proximity of NCAP sites to their homes allowed them to participate in community activities and household chores while also getting their ART. NCAP RoC also appreciated reduced transport distance, time, and costs, which consequently encouraged them to adhere to community-based appointments. While this study took place in urban settings, other RoC in DSD care models in rural settings also experience reduced transport costs compared to a traditional facility-based care model [30]. Implementing DSD care models in rural settings could have strong implications for RoC in rural settings where transport time and costs are particularly high [31]. Future work should evaluate the cost effectiveness of NCAP for RoC.

Quality of care was another important consideration in participants’ choice of care model. Clinic-based RoC reported high satisfaction with the static-site teams, which may be attributed to the comprehensive and advanced care options provided by the LT clinics, allowing easy access to integrated high-quality care in a single visit. Some participants were willing to travel long distances to get care at LT because of the quality of care, suggesting that high quality care might be an important motivation to overcome access barriers including travel time and cost [32]. Although some RoC in community-based models may fear detachment from the formal health system [12,32], NCAP RoC felt that NCAP enabled comprehensive care through referrals and reminders for other preventative healthcare made by community nurses. Even more, NCAP RoC preferred the quality of treatment they received from community nurses over that of clinic nurses, noting their friendliness and respect. Community nurses attend to few RoC overall, allowing for a more tailored and personalized care approach. RoC satisfaction with community nurses interaction also supports the potential for improving RoC-centered care with DSD models [33] that reinforce the benefits of peer support.

This study complements a previous NCAP pilot study [7] that identified the central role of peer support in supporting health and wellness among NCAP participants. NCAP RoC felt that community-based care brought individuals together for peer support, a unique benefit of NCAP, encouraging them to overcome barriers to retention. A longitudinal study of retention comparing the two care models could better examine care model impact. Peer support groups with strong leaders like in NCAP minimize the nurse workload, reflecting potential DSD advantages for both providers and RoC [34,35]. In this study, participants continued to appreciate reduced costs and improved convenience of NCAP, appeared satisfied with their care, and valued the privacy benefits afforded by community-based care. However, the previous NCAP study noted LT crowding and long waiting times as a primary reason to switch to NCAP-based care, something that was not identified in this study. NCAP expansion may have reduced LT wait times, an intended positive result of DSD scale-up.

This study also had limitations. The flexibility that is offered with NCAP participation is restricted to eligible LT RoC, not all LT clients. We engaged participants established in care and their reasons for choosing NCAP or clinic-based care are subject to choice-supportive bias, meaning that positive attributes are ascribed to the chosen option [36]. There is potential loss of nuance in participants’ contributions in translating FGDs from Chichewa to English. We tried to minimize this with the involvement of a Malawian qualitative researcher who conducted and translated the FGDs into English. The FGDs were stratified by type of DSD care model and not gender, and the sample was 81% female. Therefore, we likely lacked adequate numbers of men to explore gender difference in care preferences. Moreover, all NCAP and clinic sites where participants were recruited from were in urban settings from a Centre of Excellence. We acknowledge that the sampling could contribute to the homogeneity of our findings. Future research should explore care models in rural settings and compare DSD care models across other routine clinics in low-resource settings. Despite limitations, these findings offer useful feedback for program planners and providers.

## Conclusion

RoC are making informed choices about where to receive care, considering convenience, quality of care, protection of privacy, and potential for mutual support. The varying needs, characteristics, and preferences of RoC underscore that there is no one-size-fits-all care model, but rather that lifelong HIV care requires flexibility for RoC to choose between clinic-, community- or other DSD-type models as preferences and life circumstances change. This adaptive and responsive approach can reduce burdens and concerns that impede access to services and retention in care. While there are similar strengths between both clinic- and community-based models, peer support is a unique benefit of the community-based model that likely positively impacted retention. Future research should explore retention in both care models and appropriate DSD care models in rural settings. Although not all care options are always available to everyone, increased flexibility for care model changes and allowance for more fluid movement between models may better meet RoC expectations for convenience, privacy, and quality care, enhancing retention and satisfaction over time.
